# Socioeconomic Status and Psychosocial Resources Mediate Racial/Ethnic Differences in Psychological Health Among Gay and Bisexual Men: A Longitudinal Analysis Using Structural Equation Modeling

**DOI:** 10.1177/15579883211001197

**Published:** 2021-03-16

**Authors:** Rainier Masa, Sylvia Shangani, Don Operario

**Affiliations:** 1 School of Social Work, University of North Carolina, Chapel Hill, NC, USA; 2 School of Community and Environmental Health, Old Dominion University, Norfolk, VA, USA; 3 School of Public Health, Brown University, Providence, RI, USA

**Keywords:** socioeconomic factors, well-being, sexual and gender minorities, ethnic groups, homophobia, structural equation modeling

## Abstract

A large body of research demonstrates disparities in psychological health attributed to sexual minority identity, racial/ethnic minority identity, and socioeconomic status (SES). Fewer studies have explicated the role of these multiple attributes on psychological health and explored the role of SES and psychosocial resources in determining outcomes. We analyzed data from Project STRIDE, a longitudinal survey involving a diverse sample of gay and bisexual adult men (*n* = 198). Using structural equation modeling, we tested hypothesized direct and indirect effects of race/ethnicity, SES, and three psychosocial mediational variables (collective self-efficacy, everyday discrimination, internalized homophobia) on two outcome variables—psychological and social well-being—assessed at 1-year follow-up. Our model indicated that: (1) race/ethnicity and SES were significantly associated with each other and with each psychosocial mediator; (2) higher SES was directly and indirectly associated with both measures of well-being; and (3) collective self-esteem and everyday discrimination mediated the association between SES and both measures of well-being. The model also indicated that racial/ethnic associations with psychological mediators and outcomes are evident in the context of SES, but these effects might be suppressed when the model does not consider SES. Findings highlight the critical role of SES and race/ethnicity in determining the psychological and social well-being of sexual minority men. Specification of mediating variables—collective self-efficacy, everyday discrimination, internalized homophobia—indicates potential intervention targets to improve psychological and social health in sexual minority men. Associations between race/ethnicity and SES support the need for intersectional frameworks in addressing the health of sexual minority men.

Socioeconomic status (SES) is a well-established predictor of well-being, including physical and mental health (Moor et al., 2017; O’Brien et al., 2020). Studies have identified low SES as a consistent and robust predictor of adverse physical and mental health (Grintsova et al., 2014; Meader et al., 2016; Spencer et al., 2013). Various SES indicators indicate that a higher proportion of gay and bisexual persons have lower SES than their heterosexual peers (Badgett et al., 2013; Conron et al., 2018). For example, income poverty is higher among gay (20.5%) and bisexual (25.9%) persons than heterosexual (15.3%) men (Badgett et al., 2013). While gay and bisexual males tend to have higher education than their heterosexual peers, they are less likely to own homes, have fewer economic resources, and are at increased risk of economic hardship, or inability to pay their bills and afford food, compared to heterosexual males (Conron et al., 2018). Evidence also indicates that gay and bisexual persons are more likely to experience adverse physical and mental health compared with heterosexual individuals (Clarke et al., 2019; Elliott et al., 2015; Gustafsson et al., 2017; Logie et al., 2018). However, SES remains an understudied determinant of health for gay and bisexual persons (Ompad et al., 2018). The limited research on SES and health of gay and bisexual persons is surprising given that an established body of literature links SES to poor physical and mental health (Bleich et al., 2012; Moor et al., 2017) and indicates gay and bisexual persons at higher risk for adverse health outcomes compared to their heterosexual peers (Jackson et al., 2016; Operario et al., 2015).

In addition to SES, race/ethnicity remains a prominent factor explaining physical and mental health disparities in heterosexual and non-heterosexual populations (Krieger et al., 2003; Shangani et al., 2020). Gay and bisexual persons of color are more likely to experience adverse health outcomes and to have unmet physical and mental health needs than White gay and bisexual persons (Hsieh & Ruther, 2016; Jeong et al., 2016; Trinh et al., 2017). The interaction of race/ethnicity and SES in gay and bisexual individuals reflects the pattern observed in heterosexual populations, in which a higher proportion of gay and bisexual persons of color has lower SES compared to their non-LGB counterparts of the same race/ethnicity (Conron et al., 2018; Gates & Kastanis, 2013a, 2013b). Racial/ethnic differences in physical and mental health are maintained by various forms of economic and noneconomic discrimination (Bailey et al., 2017; Mehra et al., 2017). After accounting for individual-level SES differences, researchers have attributed the marked persistence of racial/ethnic inequities in health to racism and its adverse impact on resource distribution and stress (O’Brien et al., 2020; Williams et al., 2019; Williams & Mohammed, 2013).

Our study seeks to reframe health disparities by examining pathways that may explain the relationship between sexual and racial/ethnic minorities and psychological health. In this study, we started to investigate potential pathways that heighten risk for adverse health outcomes among individuals with multiple minority identities, including Black and Latinx GBM. This approach recognizes the intersection of sexual orientation and race/ethnicity, which has been the focus of an emerging body of literature on intersectionality (Bowleg, 2012; Hsieh & Ruther, 2016; McGarrity, 2014). Guided by developments in the minority stress model (Meyer, 2003) and intersectionality theory (Bauer, 2014; Crenshaw, 1991), researchers are examining the additive and multiplicative factors that contribute to adverse health outcomes among individuals with multiple minority identities. This research orientation allows us to shift from an ahistorical, acontextual, risk-based, and individual approach to understanding health disparities to a historical, contextual, and resilience-based approach (Volpe et al., 2019). Current intersectionality research with sexual and gender minorities, albeit limited, suggests that economic or financial disadvantage reinforces adverse health outcomes at the intersection of sexual orientation and sociodemographic characteristics (Amroussia et al., 2019). Amroussia et al. (2019) reported that inequalities in cigarette smoking at the intersection of education and sexual orientation were primarily explained by differences in levels of microeconomic resources, defined as financial resources an individual or a household receives, with income being the main contributor to inequality (Amroussia et al., 2019). Although initial studies suggest the importance of microeconomic factors, limited evidence exists to support the assertion that gay and bisexual persons of color are more likely to experience adverse health outcomes because of their SES compared to their White counterparts.

This study begins to address evidence gaps by simultaneously examining the association of SES, race/ethnicity, and well-being in a longitudinal cohort study of gay and bisexual males. We aim to investigate the direct and indirect associations of race/ethnicity with psychosocial resources and direct and indirect associations of SES with psychological and social well-being. We are interested in empirically testing the following relationships: (1) direct and indirect association of SES with well-being and (2) direct and indirect association of race/ethnicity with psychosocial resources. In this study, we assessed the indirect association of race/ethnicity via SES on psychosocial resources and the indirect effect of SES via psychosocial resources on well-being. Outcomes were assessed at 1-year follow-up. Consistent with the literature (Junker et al., 2019; Pascoe & Smart Richman, 2009), we examined whether psychosocial factors such as perceived discrimination and collective self-efficacy mediate the relationship of SES with well-being. We also evaluated mediating relationships given prior research that indicates a direct correlation of SES and psychosocial functioning, and psychosocial functioning and health outcomes (Brown et al., 2015; Matthews et al., 2010). Few studies have empirically assessed the potential mediating role of psychosocial resources. Our mediation models also allowed us to examine additional direct associations: SES and psychosocial resources, race/ethnicity and SES, and psychosocial resources and well-being.

## Methods

### Design

Data came from Project STRIDE: Stress, Identity, and Mental Health, a large National Institute of Mental Health (NIMH)-funded longitudinal research study conducted in the New York City area (Meyer et al., 2006). A detailed description of Project STRIDE methodology has been described elsewhere (Meyer et al., 2006). Baseline data were collected between February 2004 and January 2005, and follow-up data were collected a year after baseline. All interviews were conducted in person using computer-assisted and paper-and-pencil questionnaires. Project STRIDE’s study procedures were approved by a university-affiliated institutional review board. All respondents signed a written informed consent after the study procedure had been fully explained to them (Gordon & Meyer, 2007).

### Sample

Project STRIDE participants (*n* = 524) were recruited using venue-based and snowball sampling methods to ensure diversity of respondents based on gender, sexual orientation, race/ethnicity, and age. All respondents were recruited in person by research workers who approached potential study participants in the sampling venues. A cap of 25% was established for the number of respondents taken from the five venue types: bars, non-bar establishments, outdoors, groups, and events. Snowball sampling was used to recruit respondents who were less likely to be identified in these venues and to increase the diversity of the study sample. Respondents recruited in public venues were given letters of invitation to pass along to their friends and colleagues. At each venue, research staff explained the study and its activities to potential participants, who then filled out a screening form to determine eligibility (Meyer et al., 2006).

After screening for eligibility, respondents were selected using a representative quota sampling method from the pool of eligible screened individuals. Individuals were eligible to participate in the study if they (1) self-identified as cis-gender male or female and were assigned that sex at birth; (2) self-identified as lesbian, gay, bisexual (LGB), straight, or used other terms conveying such identification (e.g., queer, heterosexual); (3) self-identified as White, Black, or Latino or used other terms conveying such identifications (e.g., Hispanic, African American); (4) were between the ages of 18 and 59; (5) resided in New York City for two years or more; and (6) were able to speak English well enough to engage in casual conversation (Meyer et al., 2006). Individuals were not eligible to participate in the study if a close family member or live-in partner already participated in the study. Detailed information about the recruitment of the study sample, including a description of the sampling venues, the screening form, number of approached and eligible respondents, quota sampling method, and response and cooperation rates are available in Meyer et al. (2006). Given the aims of this paper, we restricted our analytic sample to respondents who identified as cisgender male and gay, bisexual, or homosexual, resulting in a sample of 198 participants.

### Measures

#### Socioeconomic Status

SES referred to respondents’ access to social and economic resources at baseline. We operationalized SES as a latent variable, given that there is no single best-observed indicator of SES (Duncan et al., 2002; Galobardes et al., 2006). To increase construct validity, we created a measure of SES based on available indicators that have been reported to influence health outcomes, including racial/ethnic and sexual orientation differences in physical and mental health (Duncan et al., 2002; Riley, 2018; Spencer et al., 2013; Williams et al., 1997). These indicators were measured at baseline and included education (higher than high school education or a high school education and less), household income (measured in dollars), employment status (employed or unemployed), net worth (positive, i.e., money left over after subtracting loans and debts from assets, or negative, i.e., owed money after subtracting loans and debts from assets), and two potential sources of chronic strain: finances and residence. Residence was measured with two questions, whereas finances were measured with one question. Each item asked respondents, on a scale of 1–3, to indicate whether statements such as “There are some places in your neighborhood where you do not feel safe” were not true, somewhat true, or very true for them at the time of data collection (Wheaton, 1999).

#### Well-Being

Well-being referred to two types: psychological and social. Both variables were assessed at 1-year follow-up. Psychological or personal well-being assessed the respondents’ perception of various aspects of their psychological well-being, including autonomy, environmental mastery, personal growth, positive relations with others, purpose in life, and self-acceptance (Ryff & Keyes, 1995). This outcome variable was measured using an 18-item, six-point Likert type scale ranging from 1 (strongly disagree) to 6 (strongly agree). Social well-being examined the respondents’ perception of their social environment. This outcome variable was measured using a 15-item, seven-point Likert type scale ranging from 1 (strongly agree) to 7 (strongly disagree) (Keyes, 1998). The social well-being measure included items on acceptance, actualization, contribution, coherence, and integration. For each type of well-being, we used the total well-being score collected at 1-year follow-up. The total well-being score was calculated by summing the subscale scores for each participant. Subscale scores, or the mean subscale scores for each participant, were obtained by summing individual item scores and then dividing the summed score by the number of items in the subscale (Meyer et al., 2006). Psychological well-being included six factors, whereas social well-being comprised five factors. Each subscale for both measures of well-being contained three items. Higher scores reflected higher psychological or social well-being.

#### Psychosocial Mediators

We included three types of psychosocial mediators that have been identified to correlate directly with race/ethnicity, SES, and well-being (Bamishigbin et al., 2017; Halkitis et al., 2013; Herrick et al., 2013). These mediators were everyday discrimination, internalized homophobia, and collective self-esteem. For each mediator, we used the mean total score collected at 1-year follow-up. The mean total score for each participant was estimated by summing responses to individual items and then dividing the summed score by the number of items in the scale (Meyer et al., 2006). All the scales and measures used in the analyses had good reliability (Meyer et al., 2006).

##### Everyday discrimination

Everyday discrimination referred to respondents’ experience of chronic and routine unfair treatment. Meyer et al. (2006) adapted the original scale developed by Williams et al. (1997) to ensure relevance to all minority groups in the study. This variable was measured using an eight-item, four-point Likert type scale ranging from 1 (*often*) to 4 (*never*). Items included: “How often have you been called names or insulted?” and “How often have you experienced people acting as if they are afraid of you?” Responses were recoded so that higher scores reflected the frequent experience of discrimination.

##### Internalized homophobia

Internalized homophobia evaluated the extent to which gay and bisexual males do not accept their sexual orientation, are uneasy about their same-sex desires, and seek to avoid homosexual feelings (Herek & Glunt, 1995). This variable was measured using a nine-item, four-point Likert type scale with response options ranging from 1 (*often*) to 4 (*never*). Sample items included: “How often have you wished you were not gay?” and “How often have you wished that you could develop more erotic feelings toward the opposite sex?” Responses were recoded so that higher scores indicated greater internalized homophobia.

##### Collective self-esteem

Collective self-esteem assessed respondents’ evaluation of their collective identity and group memberships (Luhtanen & Crocker, 1992). This variable was measured using a 16-item, seven-point Likert type scale ranging from 1 (*strongly agree*) to 7 (*strongly disagree*). Items included, “I am a worthy member of the social groups I belong to” and “Overall, my social groups are considered good by others.” Responses were recoded so that higher scores indicated a higher level of collective self-esteem.

#### Demographics

We included three baseline demographic variables in our structural equation model: race/ethnicity (White, Black, or Latinx), country of birth (the United States or not in the United States), and household size, defined as the number of people in the household. Country of birth and household size were added as covariates when we examined the relationship between race/ethnicity and SES.

#### Analysis

**Figure 1.**
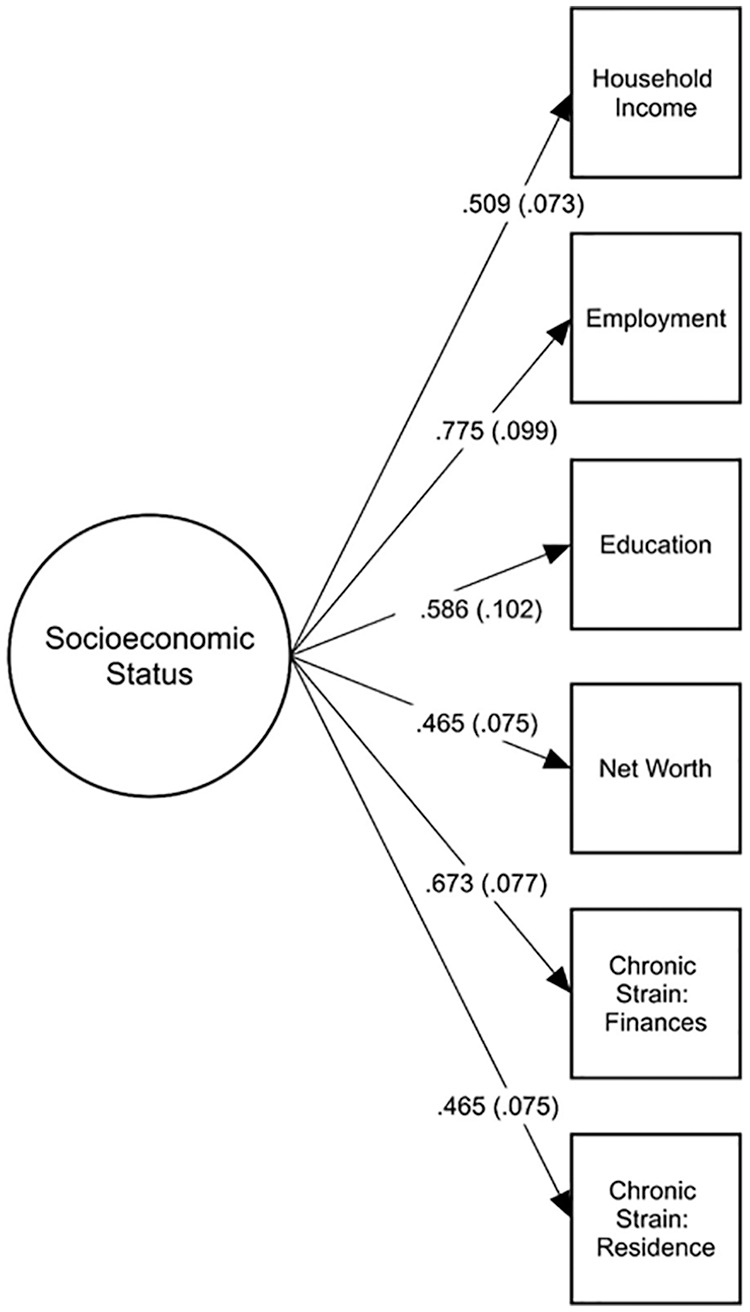
Confirmatory factor analysis model of socioeconomic status (SES). The circle represents the latent SES variable, and squares represent the six observed items. All standardized factor loadings had *p* values <.001.

Our analysis comprised two steps. First, we estimated an SES measurement model and evaluated its fit using confirmatory factor analysis (CFA). Figure 1 illustrates a visual representation of our SES measurement model. All SES indicators were measured at baseline assessment. We used CFA to determine whether our hypothesized latent SES variable adequately represented the relationship that exists in the data before estimating the structural model. The value of establishing measurement model adequacy before analyzing the structural model is widely considered a best practice (Anderson & Gerbing, 1988). We used the weighted least square mean and variance-adjusted estimator with missing values as the estimation method for both measurement and structural models (Muthén & Muthén, 2017). Our analytical sample included 29 cases with missing values. We evaluated model fit using the χ^2^ test, which is an appropriate measure of fit for models with up to 200 cases (Barrett, 2007). We also assessed model fit using additional indices such as the root mean square error of approximation (RMSEA), comparative fit index (CFI), and Tucker–Lewis index (Kline, 2016).

**Figure 2.**
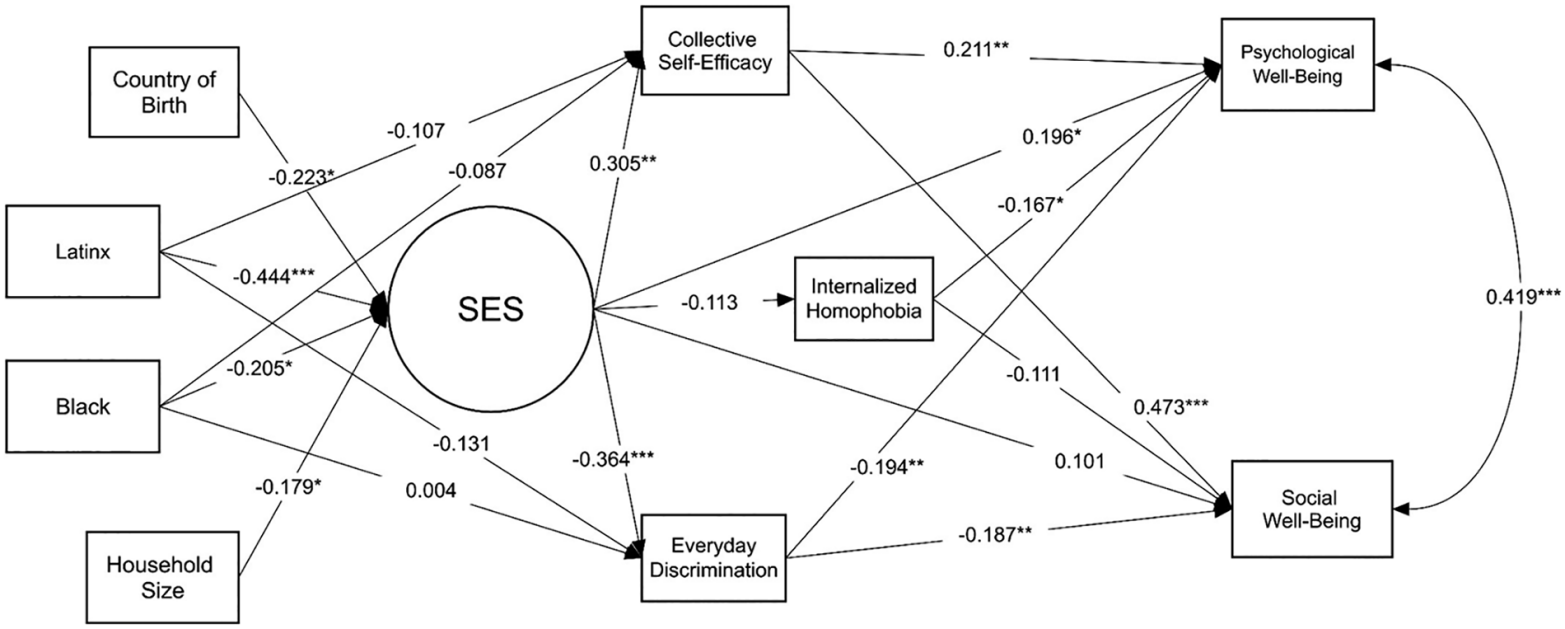
Structural equation model of our hypothesized directional relationships among SES, race/ethnicity, psychosocial resources, and well-being. *Note*. SES = socioeconomic status. *p ≤ .05, **p ≤ .01, ***p ≤ .001. SES and race/ethnicity were measured at baseline. All other variables were measured at follow-up (or 1 year later). Circle represents the latent SES variable and rectangles represent observed variables. Reference groups: born outside the United States for country of birth, non-Latinx (Black and White) for Latinx, and non-Black (Latinx and White) for Black. Not depicted: direct effect of Black (β = 0.217) and Latinx (β = 0.221) on internalized homophobia, and covariances of psychosocial resources. Covariances: collective self-efficacy and internalized homophobia = −0.310, *p* < .001; collective self-efficacy and everyday discrimination = −0.007, *p* = .93; internalized homophobia and everyday discrimination = 0.260, *p* < .001.

Second, after the SES measurement model was assessed to be adequate, we specified our structural model, which included directional relationships, based on empirical evidence reviewed in the introduction. The structural model allowed the testing of the study hypotheses, including direct and indirect associations. Figure 2 displays a visual representation of our recursive, structural model, including the hypothesized directional relationship between and among observed and latent variables. Psychosocial mediators and well-being outcomes reflected scores based on follow-up assessment. After specification and identification, we estimated the structural model and evaluated its fit. We assessed the structural model fit using the same fit indices used in the evaluation of the SES measurement model’s fit (χ^2^ test, RMSEA, CFI, and TLI). As for indicators of good fit, we used a nonsignificant c χ^2^ test (*p* > .05), CFI, and TLI of .95 (or higher) and RMSEA point estimate of .06 (or lower) and upper confidence interval of .06 or (lower) (Hu & Bentler, 1999; Kline, 2016). Given a lack of consensus on goodness-of-fit indices and recommended cutoff values for assessing fit, we used these values, taking into account the limitations noted in the literature (Chen et al., 2008; Lai & Green, 2016). All analyses were conducted using Mplus version 8 (Muthén & Muthén, 2017). The publicly available data provided summed scores for each scale, and thus Cronbach’s αs were not computed for included measures; psychometric measurement details are provided in Meyer et al. (2006). The data that support the findings of this study are openly available through the Inter-university Consortium for Political and Social Research at https://doi.org/10.3886/ICPSR35525.v2, reference number [ICPSR 35525].

## Results

### Sample Characteristics

Table 1 presents the characteristics, including the six SES indicators, of the study sample by race/ethnicity and their bivariate association with race/ethnicity. Thirty-four percent (*n* = 67) of respondents were White, 34% (*n* = 67) Black or African American, and 32% (*n* = 64) Latinx or Hispanic. Overall, 78% (*n* = 155) of the sample, regardless of race/ethnicity, identified as gay, 14% (*n* = 28) as bisexual, and 8% (*n* = 15) as homosexual. Bivariate associations indicated significant differences among White, Black, and Latinx participants on their country of birth and three of six SES indicators: employment, income, and finances as a source of chronic strain. A higher proportion of Latinx respondents (36%, *n* = 23) were born outside the United States, compared to White (10%, *n* = 7) and Black (15%, *n* = 10) respondents. A higher proportion of White respondents reported being employed (92%, *n* = 62) compared to their Black (83%, *n* = 56) and Latinx (76%, *n* = 49) peers. Similarly, a higher proportion of White gay and bisexual males (55%, *n* = 37) did not experience finances as a source of chronic strain in the lives compared to Black (39%, *n* = 26) and Latinx (23% *n* = 15) gay and bisexual males. Last, 46% (*n* = 30) of White gay and bisexual males reported earning more than $50,000 annually, compared to 16% (*n* = 10) and 28% (*n* = 19) of Latinx and Black gay and bisexual males, respectively.

**Table 1.** Sample Characteristics.

Variables	White (*n* = 67)	Black (*n* = 67)	Latin (*n* = 64)	*p*
Sexual orientation				.10
Gay	84%	73%	78%	
Bisexual	6%	18%	19%	
Homosexual	10%	9%	3%	
Place of birth				.00
United States	90%	85%	64%	
Outside the United States	10%	15%	36%	
Household size				.07
1 person	85%	61%	56%	
2 people	7%	16%	17%	
3 people	6%	10%	16%	
More than 3 people	2%	13%	11%	
Education				.16
High school diploma or less	15%	25%	28%	
More than high school education	85%	75%	72%	
Employment status				.04
Employed	93%	84%	77%	
Unemployed	7%	16%	23%	
Household income				.02
$9,999 or less	3%	16%	16%	
$10,000–$19,999	12%	9%	25%	
$20,000–$29,999	14%	14%	7%	
$30,000–$39,999	14%	18%	20%	
$40,000–$49,999	11%	15%	16%	
$50,000–$74,999	24%	16%	6%	
$75,000–$99,999	11%	9%	7%	
$100,000 or more	11%	3%	3%	
Net worth				.36
Owed money	45%	56%	57%	
Money left over	56%	44%	43%	
Chronic strain: finances				.01
Not true	55%	39%	23%	
Somewhat true	30%	36%	44%	
Very true	15%	25%	33%	
Chronic strain: residence	1.40 (0.45)	1.45 (0.48)	1.63 (0.58)	.15

### Measurement Model: SES

Results indicated good fit between our SES measurement model and observed data (χ^2^ [9, *N* = 198] = 10.90, *p* = .28, RMSEA = .033, 90% CI [.000, .090], CFI = .987, TLI = .979). Standardized parameter estimates are provided in Figure 1. All factor loadings were statistically significant (*p* < .001) and greater than .40. The percentages of variance (or *R*^2^ values) in each observed item that is explained by the measurement model ranged from .22 (chronic strain: residence) to .60 (employment). We did not conduct post-hoc modifications because of the adequate fit between the data and our measurement model.

### Structural Results

Our hypothesized structural model is described graphically in Figure 2; structural results are presented in Tables 2 and 3. Results indicated good fit of our hypothesized structural equation model (χ^2^ [68, *N* = 198] = 88.11, *p* = .05, RMSEA = .039, 90% CI [.000, .060], CFI = .984, TLI = .976). We did not conduct post-hoc modifications because of the adequate fit between the data and the model.

**Table 2.** Standardized Direct, Indirect, and Total Effects of SES, and Direct Effect of Psychosocial Resources (*N* = 198).

Effects	β	*SE*	*p*
From SES to psychological well-being			
Total effect	0.350	0.078	.000
Total indirect effect	0.154	0.047	.001
Specific indirect effect			
Via collective self-efficacy	0.064	0.028	.023
Via internalized homophobia	0.019	0.016	.247
Via everyday discrimination	0.071	0.028	.010
Direct effect	0.196	0.081	.016
From SES to social well-being			
Total effect	0.325	0.083	.000
Total indirect effect	0.225	0.059	.000
Specific indirect effect			
Via collective self-efficacy	0.144	0.046	.002
Via internalized homophobia	0.013	0.012	.295
Via everyday discrimination	0.068	0.026	.008
Direct effect	0.101	0.074	.174
From SES to collective self-efficacy			
Direct effect	0.305	0.091	.001
From SES to internalized homophobia			
Direct effect	−0.113	0.089	.202
From SES to everyday discrimination			
Direct effect	−0.364	0.086	.000
From collective self-efficacy to well-being			
Direct effect to psychological well-being	0.211	0.068	.002
Direct effect to social well-being	0.473	0.065	.000
From internalized homophobia to well-being			
Direct effect to psychological well-being	−0.167	0.074	.024
Direct effect to social well-being	−0.111	0.063	.081
From everyday discrimination to well-being			
Direct effect to psychological well-being	−0.194	0.061	.002
Direct effect to social well-being	−0.187	0.054	.001

*Note*. SES = socioeconomic status.

**Table 3.** Standardized Direct, Indirect, and Total Effects of Race/Ethnicity (*N* = 198).

Effects	β	*SE*	*p*
From Black to SES			
Direct effect	−0.205	0.093	.027
From Latinx to SES			
Direct effect	−0.444	0.094	.000
From Black to collective self-efficacy			
Total effect	−0.149	0.086	.083
Indirect effect via SES	−0.063	0.034	.067
Direct effect	−0.087	0.090	.336
From Black to internalized homophobia			
Total effect	0.241	0.083	.004
Indirect effect via SES	0.023	0.020	.244
Direct effect	0.217	0.089	.015
From Black to everyday discrimination			
Total effect	0.079	0.091	.383
Indirect effect via SES	0.075	0.038	.050
Direct effect	0.004	0.091	.962
From Latinx to collective self-efficacy			
Total effect	−0.243	0.092	.009
Indirect effect via SES	−0.135	0.049	.006
Direct effect	−0.107	0.108	.319
From Latinx to internalized homophobia			
Total effect	0.271	0.068	.000
Indirect effect via SES	0.050	0.041	.215
Direct effect	0.221	0.083	.008
From Latinx to everyday discrimination			
Total effect	0.031	0.095	.743
Indirect effect via SES	0.162	0.056	.004
Direct effect	−0.131	0.097	.178
From country of birth to SES*	−0.223	0.085	.009
From household size to SES*	−0.179	0.074	.016

*Note*. SES = socioeconomic status.

* only direct effect was tested.

#### Direct Effects

##### SES and well-being

Table 2 presents the standardized direct effect of baseline SES on well-being and psychosocial resources at 1-year follow-up, as well as the direct effect of psychosocial resources on well-being. Baseline SES was positively associated with both measures of well-being at follow-up. However, only the relationship between SES and psychological well-being was statistically significant (β = 0.196, *p* =. 02).

##### Race/ethnicity and SES

Table 3 presents the standardized direct effect of race/ethnicity on SES. Race/ethnicity was significantly associated with SES. Black gay and bisexual males had lower SES (β = −0.205, *p* = . 03) than their non-Black peers. Latinx males also had lower SES (β = −0.444, *p* < . 001) compared to their non-Latinx peers. Additionally, being born in the United States (β = −0.223, *p* = . 01) and increasing household size (β = −0.179, *p* = . 02) were associated with lower SES.

##### SES and psychosocial resources

Baseline SES was negatively associated with everyday discrimination and internalized homophobia and positively associated with collective self-efficacy, each assessed at 1-year follow-up. Higher SES at baseline was significantly associated with less frequent experience of everyday discrimination at follow-up (β = −0.364, *p* < . 001) and with higher collective self-esteem at follow-up (β = 0.305, *p* = . 001). The negative relationship between baseline SES and internalized homophobia at follow-up was not statistically significant (β = −0.113, *p* = . 20).

##### Psychosocial resources and well-being

Collective self-efficacy was positively and significantly associated with psychological (β = 0.211, *p* = . 002) and social (β = 0.473, *p* < . 001) well-being. Internalized homophobia and everyday discrimination were negatively associated with both measures of well-being. All associations were statistically significant, except for the association between internalized homophobia and social well-being. Greater internalized homophobia was associated with lower psychological (β = −0.167, *p* = . 02) and social (β = −0.111, *p* = . 08) well-being. More frequent experience of everyday discrimination was associated with lower psychological (β = −0.194, *p* = . 002) and social (β = −0.187, *p* = . 001) well-being. All psychosocial and well-being variables reflected follow-up assessments.

##### Race/ethnicity and psychosocial resources

Table 3 lists the direct effect of race/ethnicity on psychosocial resources. Black (β = 0.217, *p* = .02) and Latinx (β = 0.221, *p* = . 01) males scored higher on the internalized homophobia scale at follow-up compared to their non-Black and non-Latinx counterparts. Black (*β* = −0.087) and Latinx (*β* = −0.107) males reported lower follow-up collective self-efficacy than their non-Black and non-Latinx counterparts. The relationship between race/ethnicity and follow-up everyday discrimination differed. Black (β = 0.004) males reported more, albeit marginal, everyday discrimination than their non-Black counterparts. Latinx (β = −0.131, *p* = .18) males reported less everyday discrimination than their non-Latinx counterparts. The relationship of race/ethnicity with internalized homophobia at follow-up was the only significant direct association between race/ethnicity and psychosocial resources.

#### Indirect Effects

##### SES and well-being via psychosocial resources

Table 2 presents the standardized indirect effect of baseline SES on 1-year follow-up well-being via three measures of psychosocial resources. SES had a significant (total) indirect effect on psychological well-being (β = 0.154, *p* = .004). Higher SES was associated with higher psychological well-being via collective self-efficacy (β = 0.064, *p* = .02), internalized homophobia (β = 0.019, *p* = .25), and everyday discrimination (β = 0.071, *p* = .01). Additionally, SES had a significant (total) indirect effect on social well-being (β = 0.225, *p* < .001). Higher SES was associated with higher social well-being via collective self-efficacy (β = 0.144, *p* = .002), internalized homophobia (β = 0.013, *p* = .29), and everyday discrimination (β = 0.068, *p* = .01). In both models, collective self-efficacy and everyday discrimination were statistically significant indirect pathways. For both measures of well-being, collective self-efficacy had the largest predictive validity, as illustrated by the magnitude of the regression coefficient.

##### Race/ethnicity and psychosocial resources via SES

Table 3 presents the standardized indirect effect of race/ethnicity on follow-up psychosocial resources via baseline SES. Overall, baseline SES had an indirect effect that may explain the relationship between race/ethnicity and follow-up psychosocial resources. The indirect effect of SES was larger and consistent in Latinx males compared to their Black counterparts. For example, among Black gay and bisexual males, the indirect effect of baseline SES on follow-up everyday discrimination was 0.096 (*p* = .06). Among Latinx gay and bisexual males, the indirect effect of baseline SES on follow-up everyday discrimination was 0.211 (*p* = .01). Similarly, the indirect effect of baseline SES on collective self-efficacy at 1-year follow-up was −0.063 (*p* = .07) for Black males and −0.135 (*p* = .01) for Latinx males. Among Latinx males, the indirect effect of baseline SES on collective self-efficacy (β = −0.135, *p* = .01) and everyday discrimination (β = 0.162, *p* = .004), both measured at 1-year follow-up, was larger than the direct effect of being Latinx on collective self-efficacy (β = −0.107, *p* = .32) and everyday discrimination (β = −0.131, *p* = .18). Also, none of the direct associations of Latinx ethnicity with psychosocial factors was statistically significant. While race/ethnicity might not have a significant direct association with psychosocial factors, SES is an important third variable to examine. The addition of SES as a third variable appears to explain the relationship, thus giving us a fuller picture of how the relationship between race/ethnicity and psychosocial resources can be better understood and improved.

##### Suppression effects

Although most of the indirect relationships we evaluated illustrated mediation, three significant indirect associations are considered suppressor effects (MacKinnon et al., 2000; Rucker et al., 2011). Suppression occurs when the addition of a mediating or third variable increases the magnitude of the relationship between the independent and dependent variables (MacKinnon et al., 2000). In our three mediator models that examined the association of SES and social well-being, the indirect effect of SES on social well-being as mediated by collective self-efficacy was larger (β = 0.144, *p* = .002) and statistically significant compared to the direct effect of SES on social well-being (β = 0.101, *p* = .17). Another suppressor effect was illustrated by the indirect effect of being Latinx on collective self-efficacy as mediated by SES (β = −0.135, *p* = .01), which was larger and statistically significant compared to the direct effect of being Latinx on collective self-efficacy (β = −0.107, *p* = .32). The third suppressor effect occurred when we examined the association of being Latinx and experience of everyday discrimination. While the direct association of Latinx and everyday discrimination was negative and not significant (β = −0.131, *p* = .18), this relationship became positive and significant when SES was added as an indirect pathway (β = 0.162, *p* = .004). This type of suppressor effect is also called an incomplete mediation in which the direct and mediated effects have the opposite signs (MacKinnon et al., 2000). In other words, the variance that being Latinx shares with SES became a positive and significant predictor (indirect effect) of everyday discrimination. In turn, these two effects canceled each other out, resulting in a marginal or near zero total effect of being Latinx on everyday discrimination (β = 0.031, *p* = .743).

## Discussion

The primary goal of this study was to examine the direct and indirect associations of race/ethnicity with psychosocial resources and SES with psychological and social well-being. Structural equation modeling results indicated that SES is an essential determinant of psychosocial resources and psychological well-being for gay and bisexual men. Higher SES directly predicted higher collective self-efficacy, lower everyday discrimination, and both higher psychological as well as social well-being 1 year later. These results are in line with previous findings that indicate SES is a key determinant of health outcomes at the population level, and that higher SES is protective against health problems associated with being a racial or sexual minority (Pachankis et al., 2018; Thomeer, 2013). For example, Pachankis et al. (2018) identified that gay and bisexual men of higher SES report significantly lower levels of anticipated stigma compared to those of lower SES. However, there have been a few studies reporting conflicting results. Specifically, studies have been mixed about the effects of SES on African Americans’ health outcomes (Ríos-Salas & Larson, 2015; Shangani et al., 2020; Stepanikova & Oates, 2017). Stepanilova and Oates (2017) reported that everyday discrimination among African American patients was higher among those with higher levels of education and income, whereas non-Latinx White patients reported the opposite pattern. Our study adds substantially to the literature by indicating that over time, SES is critical in the psychological and social health of sexual minority men of color, and that this pathway is influenced by collective self-efficacy, everyday discrimination, and (to a lesser degree) internalized homophobia.

Our findings also suggest that race/ethnicity plays an important role beyond SES in psychosocial resources. Black and Latinx gay and bisexual men reported higher internalized homophobia compared to their non-Black and non-Latinx counterparts. Also, Black and Latinx men reported lower collective self-efficacy at follow-up. These findings align with previous studies that demonstrate poor health outcomes among sexual minority people of color (Gamarel et al., 2012; Tuthill et al., 2020). Our study provides a better understanding of the association of race/ethnicity and psychosocial resources among sexual minority individuals, that is, through SES. Additionally, we identify a complicated relationship between race/ethnicity and SES. Our results indicate that SES is an important third variable through which race/ethnicity affects psychological resources among Latinx and Black gay and bisexual men. Specifically, we confirm that the association between race/ethnicity and psychosocial resources is mediated by SES. Latinx ethnicity was not directly associated with psychosocial factors. However, it was significantly and indirectly associated with all the three indicators of psychosocial resources (everyday discrimination, collective self-efficacy, and internalized homophobia) through SES. Our results also indicated that Black race/ethnicity was significantly and indirectly associated with one psychosocial indicator (everyday discrimination) through SES. These findings highlight the importance of research aimed at increasing the understanding of the causal mechanisms behind racial/ethnic and SES disparities in health outcomes among sexual minority men of color. As noted in the literature, Black individuals in general experience poorer health outcomes compared to non-Black individuals. We extend the literature by identifying that both race/ethnicity and SES are important factors in assessing the mental health outcomes of sexual minority people of color.

Our results provide support for the role of race/ethnicity as well as SES in influencing psychosocial and social well-being. However, results also emphasize the need for further research to understand the upstream determinants and mechanisms accounting for the association between Black identity and internalized homophobia, as this was not mediated through SES. Considering the strong relationship between race/ethnicity and SES, these results provide additional support for prospective studies and interventions in nationally representative and diverse populations. Our study confirms that sexual minority people of color experience poorer psychological health outcomes, and these outcomes are mediated through SES. Our results may also have important implications for clinical trials and epidemiologic studies because different study endpoints may be differentially influenced by race/ethnicity. Our study finds that SES indicators may account for the relationship between race/ethnicity and psychosocial factors. This result is important given increased research/findings indicating health disparities among people of color but does not further contextualize these findings in terms of socioeconomic differences among racial/ethnic and sexual minorities.

Critical to the interpretation of our findings is the recognition that SES is rooted in the sociopolitical history of race in the United States. In our operationalization of SES, we incorporated indicators, such as education, employment, chronic strain due to neighborhood conditions and net worth, that are considered as downstream consequences of institutionalized racism (Williams et al., 2019; Williams & Mohammed, 2013). The construction of this variable is consistent with a more complex, multifaceted, and contextualized approach to scholarship on race, racism, and health as articulated in Volpe et al. (2019).

A major strength of this study is the use of multiple SES indicators measured at the individual level. We used education, income, employment status, net worth residence, and finances. Also, the study adopted a longitudinal design, assessing measures at baseline and after a 1-year follow-up. Lastly, the study sample was diverse in terms of age, SES, and race/ethnicity, drawn from New York City neighborhoods. However, some important limitations in the current study should be noted. Methodologically, the self-reported nature of our data precludes drawing causal inferences from our findings because of recall or response biases. Also, mediation, as described in the current study, was in the statistical sense only; replication with prospective measurement of the mediators and outcomes would be needed to validate these models. Along these lines, the estimate of a third variable effect is subject to sampling variability, and as a result, in any given sample, a variable may appear to act as a mediator, confounder, or suppressor only due to chance (MacKinnon et al., 2000). Also, the study design included only two assessments. While temporal order was established between baseline SES and follow-up mediator and outcome variables, temporal order between mediators and outcomes was not evident as both variables were collected at follow-up. From a study sample perspective, although a major strength of our study is its representation of a diverse group, this sample representation may also limit the generalizability of our findings to gay and bisexual men in other geographic settings in the United States or international contexts. Lastly, the data for this study were collected between 2004 and 2005 and might not represent the current trends in the associations between SES, race/ethnicity, and psychological health. In light of the timing of data collection, additional research is needed to assess whether these patterns of association have changed over time. If patterns have changed since the timing of data described here, further research is necessary to examine macro-level or individual-level factors that account for differences in these associations.

In conclusion, our findings highlight the critical role of SES and race/ethnicity in the psychological and social well-being of sexual minorities. These findings correspond with and extend intersectional frameworks, which have underscored sexual minority status and race/ethnicity as interactive, mutually constitutive factors that create various types of discrimination and oppression, which in turn, determine outcomes and inequities in health. Specifically, this analysis brings attention to SES as an often under-conceptualized and invisible third variable that, alongside sexual minority status and race/ethnicity, is crucial for intersectional frameworks on health. Therefore, interventions to address health inequities among sexual minorities must consider SES as an organizing concept that shapes people’s lived experiences, exposure to stress, and resources for coping with stress. Moreover, the findings reported here indicate the importance of psychological resources (collective self-esteem, everyday discrimination, internalized homophobia) as targets for guiding psychosocial or public health interventions with these intersectional populations. Given the observed differences between Black and Latinx gay and bisexual men, culturally informed approaches are necessary to consider how to enhance these psychological resources to improve the health of these distinct groups.
